# Characterization and Antifungal Activity of Pullulan Edible Films Enriched with Propolis Extract for Active Packaging

**DOI:** 10.3390/foods11152319

**Published:** 2022-08-03

**Authors:** Małgorzata Gniewosz, Katarzyna Pobiega, Karolina Kraśniewska, Alicja Synowiec, Marta Chaberek, Sabina Galus

**Affiliations:** 1 Department of Food Biotechnology and Microbiology, Institute of Food Sciences, Warsaw University of Life Sciences-SGGW (WULS-SGGW), Nowoursynowska 159, 02-776 Warsaw, Poland; katarzyna_pobiega@sggw.edu.pl (K.P.); karolina_krasniewska@sggw.edu.pl (K.K.); alicja_synowiec@sggw.edu.pl (A.S.); marta.s.chaberek@gmail.com (M.C.); 2 Department of Food Engineering and Process Management, Institute of Food Sciences, Warsaw University of Life Sciences-SGGW (WULS-SGGW), Nowoursynowska 159, 02-776 Warsaw, Poland; sabina_galus@sggw.edu.pl

**Keywords:** film, natural polymer, pullulan, propolis, antifungal activity, active packaging

## Abstract

Active pullulan films with the addition of 3, 5 or 10% propolis extract produced by the casting method were tested in the study. Propolis extracts from Bochnia County, Siedlce County and Ełk County (Poland) were used. The appearance of the films was characterized, as well as physical parameters (thickness, moisture content, water solubility), tensile strength (TS), elongation at break (EB), optical characteristics (light transparency, UV barrier, color) and antifungal properties. The antifungal activity of the films was tested by the disc diffusion method against yeast (*Candida albicans*, *C. krusei*, *Saccharomyces cerevisiae*, *Rhodotorula mucilaginosa*) and mold (*Alternaria solani, Fusarium solani*, *Rhizopus stolonifer*, *Colletotrichum gloeosporioides*, *C. cladosporioides*, *Aspergillus niger*, *A. ochraceus*, *Mucor mucedo*, *Penicillium expansum*, *P. chrysogenum*). The origin of propolis influenced the color and water solubility of the films. The addition of increasing concentrations of propolis extract increased the film thickness and the intensity of the yellow color, extended the water dissolution time of the film and reduced the values of TS and EB. The addition of propolis extract in the pullulan film improved UV radiation protection but decreased light transparency. The antifungal activity increased significantly with the increasing concentration of propolis extract in the film, regardless of the origin of propolis. Molds showed greater sensitivity to pullulan films containing propolis extract than yeasts. In general, films made of pullulan with the addition of propolis extract can be considered as natural active packaging to protect against the growth of fungi in food.

## 1. Introduction

Protecting food against the effects of external factors is the basic function of food packaging. The increase in demand for safer and healthier food contributed to the development of a new concept in food preservation called active packaging [1]. Active packaging is a system in which the product, packaging and the environment have a positive effect, improving safety and sensory properties and maintaining product quality, which contributes to a longer shelf life [2,3]. In order to add antimicrobial activity to edible films, antimicrobials (acids, their salts and anhydrides, nisin, antibacterial enzymes, plant extracts and essential oils) are incorporated into the matrix [4,5,6]. Edible films containing antimicrobials can prevent food spoilage caused by microorganisms, including fungi [7]. The development of fungi in food generates huge food losses [8,9]. Fungi and their toxic metabolites such as mycotoxins are among the major contaminants in food, which enter the food chain during plant cultivation, food processing, storage, and transportation. Food contaminated with fungi is unfit for human and animal consumption [10]; therefore the restriction of yeast and mold growth in food continues to be a high priority for the food and agricultural industries [11,12,13].

Pullulan is a polysaccharide produced extracellularly by the fungus *Aureobasidium pullulans* [14,15]. Pullulan molecule is composed of α-trisaccharide units linked by an α (1 → 4) glycosidic bond. In turn, α-trisaccharide units are polymerized many times with α (1 → 6) glycosidic bonds. The interest in the use of pullulan in food technology, as well as in biomedicine and pharmacy, is growing due to several advantages, the most important of which is the lack of taste and odor. Pullulan is also harmless to the human organism [16,17,18,19]. This polysaccharide has very good film-forming properties. Pullulan films are transparent, colorless and shiny with relatively low oxygen and lipid permeability. Additionally, they are amenable to changes in mechanical and gas barrier properties on mixing with biopolymers and plasticizers [18,20,21]. The incorporation of active chemicals into pullulan is a subject that has been explored more and more in recent years. Pullulan films are considered as carriers for active substances, such as antimicrobials, and have the potential to play a role in active food packaging [22,23,24,25,26].

In recent years, there has been growing interest in research into the use of edible films containing natural preservatives in food protection. One of them is propolis, which is recognized all over the world as a natural product rich in bioactive chemical components [27]. Propolis is a resinous material collected by bees (*Apis mellifera* L.) from plant buds and exudates, then mixed with bee enzymes, pollen and wax [28]. The main plant source of European propolis is poplar, especially black poplar (*Populus nigra*), and therefore it is called poplar propolis [29]. Among these organic compounds, chemical analysis of propolis has pointed to the presence of phenolic compounds and esters, flavonoids, terpenes, β-steroids, aromatic aldehydes and alcohols, sesquiterpenes, and stilbene terpenes [30,31]. In food technology, the use of propolis extract as a natural preservative has been considered [30,32,33,34,35,36,37,38,39,40,41]. Propolis extracts inhibit the growth of food contaminating fungi and can replace synthetic fungicides [42]. The action of propolis extract against various molds and yeast from the genera *Aspergillus, Fusarium, Penicillium, Botrytis, Colletotrichum, Candida, Saccharomyces, Rhodotorula, Kloeckera* and *Cryptococcus* have been demonstrated [41,42,43,44]. The composition of propolis varies with different environmental factors, e.g., the season of the year, or the source of the substance used for its production [28,33], which may affect the antimicrobial activity of propolis extracts. In our previous studies, we demonstrated the antimicrobial effect of pullulan coatings with propolis extracts from Toruń County (Poland) on cherry tomatoes [45] and blueberries [46].

The aim of this study is the physical, mechanical and optical characterization of produced pullulan-based films containing different concentrations of ethanol extracts of propolis from three regions of Poland and to investigate the antifungal potential of these films against yeast and molds causing food spoilage.

## 2. Materials and Methods

### 2.1. Materials

Food grade pullulan (99% purity) was purchased from Hayashibara Co. (Okoyama, Japan). Three ethanol propolis extracts, obtained and analyzed by HPLC-DAD, as described by Pobiega et al. [30], from Bochnia County (E1), Siedlce County (E2) and Ełk County (E3) were used. Propolis samples after freezing at −20 °C were mechanically ground. Then 100 g of a sample of powdered raw propolis was extracted with a 10-fold volume of 70% ethanol solution. Subsequently samples were subjected to ultrasound (Omni Ruptor 4000, OMNI International the Homogenizer Company, Kennesaw, GA, USA). Sonication was carried out in an ice-water bath at 210 W power and 20 kHz frequency for 20 min. The samples were then shaken at 200 rpm for 1 day at 28 °C (SM-30 Control, Edmund Bühler, Germany). The extracts were filtered through a Whatman filter paper no 4 (Millipore Merck, Darmstadt, Germany), then condensed under reduced pressure at 40 °C (Rotavapor R-215, Büchi, Flawil, Switzerland) by evaporation of the solvent and lyophilized. Sample propolis extracts were stored at 4 °C.

In their general chemical composition, propolis extracts contained total phenols: (E1) 14,773.30 mg/100 mL, (E2) 8836.22 mg/100 mL and (E3) 8575.12 mg/100 mL; total flavonoids: (E1) 10,196.09 mg/100 mL, (E2) 4464.69 mg/100 mL and (E3) 3705.10 mg/100 mL; and total phenolic acids: (E1) 4577.21 mg/100 mL (E2) 4371.53 mg/100 mL and (E3) 4870.02 mg/100 mL, respectively [30]. Glycerol, 70% ethanol and NaCl were purchased from AlChem (Toruń, Poland) and were of analytical grade. Sabouraud Agar (SA) was purchased from Merck (Darmstadt, Germany).

### 2.2. Preparation of the Pullulan Films with Propolis Extracts

Film-forming aqueous pullulan solutions containing propolis extracts in three concentrations, 3.0, 5.0 and 10% *v/v*, were prepared using the solution casting method. These three concentrations of propolis extract were chosen based on our preliminary research (unpublished data) and literature [47]. The solution of 20 g of pullulan and 2 g of glycerol (as a plasticizer) in 200 mL of distilled water was heated at 80 ± 2 °C. Then, the ingredients were dissolved by stirring for 15 min. The defoaming of film-forming solutions was made by using a MDK-3 ultrasonic cleaner (MKD Ultrasonic, Stary Konik, Poland). After that, the solutions were cooled to room temperature, propolis extracts were added and stirring continued until complete dissolution (15 min). The control pullulan film-forming solution did not contain propolis extract. The film-forming solutions of 10 mL were poured into 90 mm petri dishes, which were allowed to reach room temperature for 48 h. After drying, the films were conditioned at 25 °C at relative humidity (RH) 55 ± 2% for 48 h. All films were tested in air-conditioned laboratories at 22–23 °C and RH = 55 ± 2%.

### 2.3. Physical and Mechanical Characteristics of Films

#### 2.3.1. Thickness

The film thickness was measured with a digital micrometer (BYK-Gardner GmbH, Geretsried, Germany) to the nearest 0.001 mm. The thickness measurements were taken of each film in at least five random positions, and a mean value was used in the calculations.

#### 2.3.2. Moisture Content

2 cm × 2 cm square film samples were weighed before (W_1_) and after dry (W_2_) at 105 °C to attain to a constant weight. The moisture content (MC) of films was calculated using the formula:

MC = (W_1_ − W_2_)/W_1_ × 100

#### 2.3.3. Water Solubility

2 cm × 2 cm square film samples were immersed in 50 mL of deionized water at a temperature of 25 ± 2 °C. While the samples were stirred with a magnetic stirrer, the time (in seconds) required for complete dissolution of the films was measured [48].

#### 2.3.4. Mechanical Characteristics

Tensile strength (TS, MPa) and elongation at break (EB, %) of the films were determined using a Texture Analyzer TA.XT2i (Stable Micro Systems, Haslemere, UK) according to the ASTM D882-02 method [49]. Prior to the measurement, films were cut into strips (70 mm × 20 mm) and mounted between self-tightening tensile grips. The initial distances of separation and velocity were adjusted to 15 mm and 1 mm/s, respectively, considering conditioned samples with the cell load of 5 kg. Mechanical properties were calculated using the average thickness of each film sample. The measurement was performed at least in triplicate.

### 2.4. Optical Characteristics of Films

#### 2.4.1. Transparency and UV-Barrier Properties

Optical properties of films were determined using a Shimadzu UV–VIS 3101-PC spectrophotometer. Rectangular pieces of film were placed directly into the test chamber of the spectrophotometer. The data were recorded by Thermo Insight software. The absorbance was measured at a wavelength of 600 nm and calculated using the following formula:

Transparency 600 = Abs_600_/L

where Abs_600_ was the absorbance value at 600 nm and L was the film thickness (mm). The UV-barrier properties were evaluated by measuring the transmittance through the prepared edible films at 280 nm. Three repetitions were taken for each film sample.

#### 2.4.2. Color

The color of the films was determined using a CR-400 model colorimeter (Minolta, Tokyo, Japan) in the CIELAB color system (L*—brightness, a*—green to red color, b*—blue to yellow color). The reference material was a standard white plate with color parameters 

L* = 92.22, a* = −0.52, b* = 0.60. The measurement for each film was performed in at least five repetitions. The total color difference (ΔE) was calculated using the formula:

$$∆E\;=\sqrt{{\left(L^{*}-L\right)}^{2}+{\left(a^{*}-a\right)}^{2}+{\left(b^{*}-b\right)}^{2}}$$

where: L*, a*, b*—values for white standards, *L*, *a*, *b*—values for the films.

### 2.5. Antifungal Activity Assay

#### 2.5.1. Fungal Test Strains and Their Preparation

The following strains of yeast were used in the research: *Candida albicans* ATCC 10231, *C. krusei* ATCC 14243, *Saccharomyces cerevisiae* ATCC 9763, *Rhodotorula mucilaginosa* ATCC 66034 and mold: *Fusarium solani* ATCC 36031, *Aspergillus ochraceus* KKP 124, *Aspergillus niger* ATCC 9142, *Penicillium chrysogenum* ATCC 10136, *P. expansum* KKP 774, *Rhizopus stolonifer* ATCC 14037, *Mucor mucedo* ATCC 38694, *Alternaria solani* ATCC 16022, *Colletotrichum gloeosporioides* DSM 62146, *C. cladosporioides* ATCC 16022. Cultures were provided by the Department of Food Biotechnology and Microbiology (WULS-SGGW, Poland). Frozen cultures in 20% glycerol at −80 °C were activated on SA at 28 ± 2 °C for 48 h (yeast) and 5 days (molds). Then, the yeast strains were grown on SA at 28 ± 2 °C for 24 h. The mold strains were grown at SA at 22 ± 2 °C for 14 days. Mold spores were collected from the well-grown mycelium. A sterile physiological solution (0.85% NaCl) was used to prepare fungal suspensions at the concentration of 1 × 10^6^ spores/mL. Yeast cells and spores were counted using a hemocytometer.

#### 2.5.2. Disc Diffusion Method

Inocula of fungal strains (1 × 10^6^ CFU/mL) were applied to the surface of the SA and left for 10 min at room temperature. Discs with a diameter of 6 mm were cut from the films using a circular knife. Then, 3 film discs were placed on the SA surface. The plates were incubated at 28 °C for 72 h. The diameters of the zones of growth inhibition (DZGI) of the test strains were measured with a caliper (without subtracting the diameter of the disc). The result is given in mm ± SD. The sensitivity of the strains to the film was classified based on the DZGI size according to the following scale: not sensitive for diameters < 8 mm; sensitive for diameters 9–14 mm; very sensitive for diameters 15–19 mm; and extremely sensitive for diameters > 20 mm [50,51].

### 2.6. Statistical Analysis

The obtained results were statistically analyzed in the Statistica 13.3 program. by StatSoft (TIBCO Software Inc., Tulsa, OK, USA). One-way ANOVA was performed. The significance of differences between the mean values was verified with Tukey’s test (*p* < 0.05). The data were expressed as the mean ± SD (standard deviation). Heatmaps, Pearson’s rank correlation analysis (*p* < 0.05) results, and plots were obtained using the R platform.

## 3. Results and Discussion

### 3.1. Appearance of Pullulan Films with Propolis Extract

**Figure 1:**
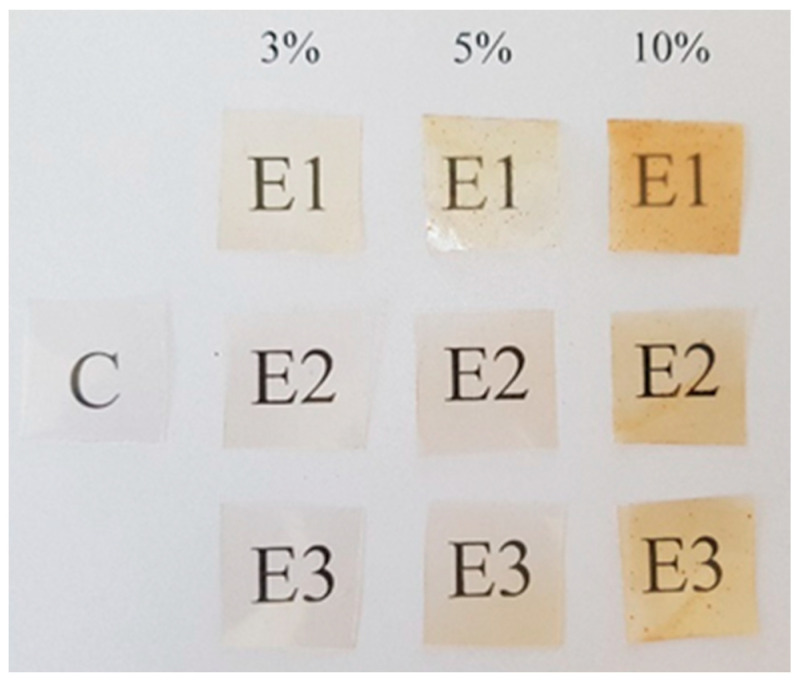
Appearance and transparency of pullulan film with propolis extract. Control pullulan film (C), pullulan films: with extract from Bochnia County propolis (E1); with extract from Siedlce County propolis (E2); with extract from Ełk County propolis (E3).

The appearance of pullulan films containing extracts of propolis and control film is shown in Figure 1. Pullulan is a polysaccharide with good adhesive properties due to the alternating repeated α (1 → 4) and α (1 → 6) glycosidic bonds in the pullulan molecule [16]. The films had a homogeneous and transparent appearance and detached easily from the surface of the Petri dishes after drying. The films were smooth, shiny, and not sticky. The control film (C) was completely without color, while the films with propolis extracts (E1, E2 and E3) at three concentrations had a yellow-orange color. However, the color intensity depended on the type of propolis extract and its concentration. The values of the color parameters of the films are presented in Table 1. The brightness of the films with propolis extracts changed slightly compared to the control film, which is indicated by the values of the L* parameter. Similarly, the values of the a* parameter slightly decreased compared to the control film. The biggest changes were in the values of the b* parameter. The more propolis extract there was in the film, the more intense was the yellow or orange hue of the films. A statistically significantly greater (*p* < 0.05) total color change (∆E) of films with E1 ranging from 3.11 to 9.16 was found than for pullulan films with E2 and E3. E1 had 2.5 times more flavonoids than E2 and over 3 times more than E3 (Section 2.1), which may be the reason for the more intense orange-yellow tinge of films with the addition of E1.

### 3.2. Thickness, Moisture Content and Water Solubility of Films with Propolis Extract

The film thickness of the film influences the mechanical and optical properties. According to Galus and Lenart [52], the thickness of the film is directly dependent on the method of preparation and drying conditions, as well as the composition and additives introduced to the film matrix. The control film had a thickness of 86.0 ± 8.5 µm, while the film samples with propolis extracts showed a greater thickness, ranging from 102.0 ± 18.2 to 248.8 ± 19.8 µm (Table 2). It was found that a higher concentration of propolis extract thickens the film. The films containing 10% propolis extract had statistically significantly greater thickness (*p* < 0.05) compared to films with 3 and 5% propolis extract (regardless of the type of propolis), which was caused by the increased content of solids in the films. The addition of meadowsweet flower extracts to the pullulan film worked in a similar manner [53].

The moisture content of the pullulan films with propolis extracts is presented in Table 2. The obtained results showed that the films containing propolis extracts had a moisture content similar (*p* > 0.05) to the control film, which is expected in films with a low content of propolis extract and plasticized with glycerol [54]. Similar results were obtained by Pastor et al. [55], who found that the addition of propolis extract below 10% had no effect on the moisture content of hydroxy-propyl-methylcellulose (HPMC) based films. The authors believe that, with a low content of propolis extract, the water sorption is mainly due to the polymer matrix and there seem to be no significant interactions with the components of the propolis extract. In contrast, a significant increase in the moisture content of the films was found when beeswax [48] was added to edible films. The beeswax addition caused water molecules to be trapped in the biopolymer matrix during the drying of emulsion films [56,57].

**Figure 2:**
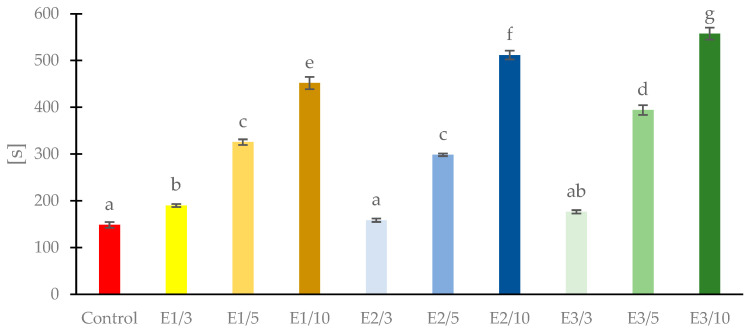
Time of water solubility of pullulan films with propolis extract. Different letters (a–g) above the columns indicate significant differences (*p* < 0.05).

Solubility is a property that determines the water resistance of edible films, which is taken into account when selecting films for specific applications [58]. The high water solubility of the film is an advantage when applying edible coatings to fresh and minimally processed foods [58,59]. Figure 2 shows the time of complete dissolution of the films in water. It was found that the solubility of the films decreased with the increase of propolis extract content in the films. The smallest (3%) addition of propolis extract had no statistically significant (*p* > 0.05) effect on the solubility of the films, but the 5 and 10% additives statistically significantly (*p* < 0.05) decreased the solubility of the films. Of the three propolis extracts, E3 had a greater effect on reducing water solubility of film than E1 and E2. Propolis is slightly soluble in water [60]. The presence of polyphenolic components in propolis extracts reduced the solubility of the films in water. Polyphenolic components of propolis, e.g., (+)−catechin, caffeic acid phenethyl ester, present very low solubilities [61,62], possibly due to strong interaction with the film network through hydrogen bonding, which reduces the affinity of the films to water and reduces the solubility of the films. According to Jouki et al. [63] the content of glycerol in the edible film has a great influence on their physical characteristics. It was observed that increasing glycerol concentration in quince seed mucilage film increased the water solubility and moisture content of those films.

### 3.3. Mechanical Properties of Films with Propolis Extract

Tensile strength (TS) and elongation at break (EB) values of pullulan films with propolis are presented in Table 2. Control pullulan films had the highest value of TS (24.62 ± 2.12 MPa) and EB (21.00 ± 0.92%) compared to the films containing propolis extracts. The addition of propolis extracts changed the mechanical properties of films. It was noted that increasing the concentration of extract in film decreases the values of TS and EB. The origin of propolis had no significant effect on the mechanical properties of the films with propolis extract. The results of this study are in line with the observation made by Pastor et al. [55] and Bodini et al. [47], who analyzed the mechanical properties of hydroxypropyl methylcellulose and gelatin film enriched with propolis extracts. Incorporation of propolis extracts into gelatin film or hydroxypropyl methylcellulose film significantly affects the tensile strength value and elongation at break value. Tensile strength was decreased with the increasing concentration of propolis extracts in gelatin films, and elongation at break was decreased in hydroxypropyl methylcellulose film. Undoubtedly, the observed changes in the mechanical parameters of films were caused by the incorporation of extracts, which act as a dispersed phase and thus produce areas of discontinuity, therefore reducing the time of the film matrix to fracture. Furthermore, Pastor et al. [55] also noted that different components of propolis extract, usually with a polar characteristic, may interact with a polymer and create a crystalline zone that decreases film flexibility and stretchability, which in turn reduce the elongation at break. General, the mechanical properties of films are dependent on their components. For example, increasing concentration of oregano essential oil (1, 1.5 and 2%) in quince seed mucilage-based films decreased the tensile strength (TS) of those films while elongation at break (% EB) increased it [6].

### 3.4. Light Barrier Properties of Pullulan Films with Propolis Extract

**Figure 3:**
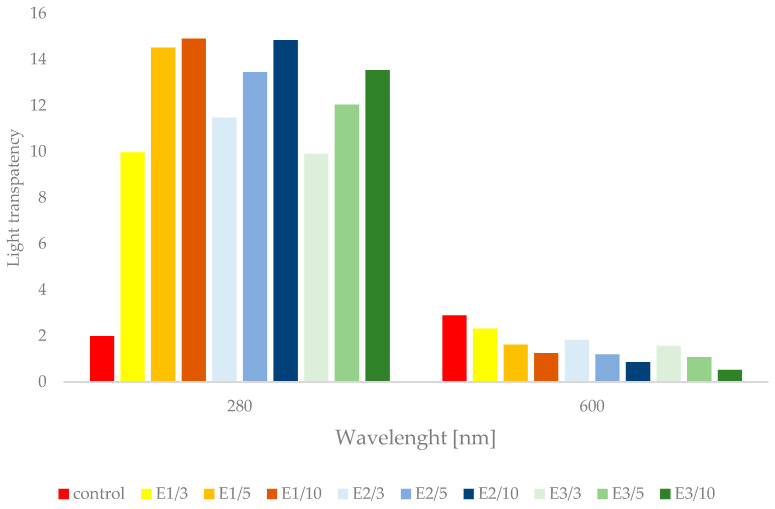
UV and light transparency of pullulan films containing propolis extracts.

The results presented in Figure 3 show that the absorbance at 280 nm of films with propolis extracts was several times higher (9.97–14.84) compared to the control film (1.99), thanks to which films with propolis extract provide good barrier properties of the pullulan film against UV light. It was found that the absorption of UV light was higher with the increase of propolis extract concentration in the pullulan film. Similar results have been reported for other edible films with the addition of extracts rich in polyphenolic compounds, e.g., tuna-fish gelatin films with the addition of murta extract [64], films based on pullulan and carboxylated cellulose nanocrystal incorporated with tea polyphenol [22] and films based on starch/polyvinyl alcohol incorporated with beta-lain-rich red pitaya (*Hylocereus polyrhizus*) peel extract [65].

The light transparency is an important feature of a film that enables the consumer to view the packaged product. The films with high consumer acceptability are colorless, transparent and do not change the color of the coated food [66,67]. In this study the control film had higher transparency (2.89) compared to other tested samples, which ranged from 0.53 to 2.31. Incorporation of propolis extracts into pullulan film decreases their transparency. Furthermore, lower transparency was noted for films with the highest addition of propolis extract. A similar observation was also made by Kumar et al. [68], who found that the transparency of chitosan film was strongly dependent on the concentration of pomegranate peel extract in film. The changes in the transparency are due to incorporation of plant extracts, which are a natural source of colorants, into the film matrix [69].

**Figure 4:**
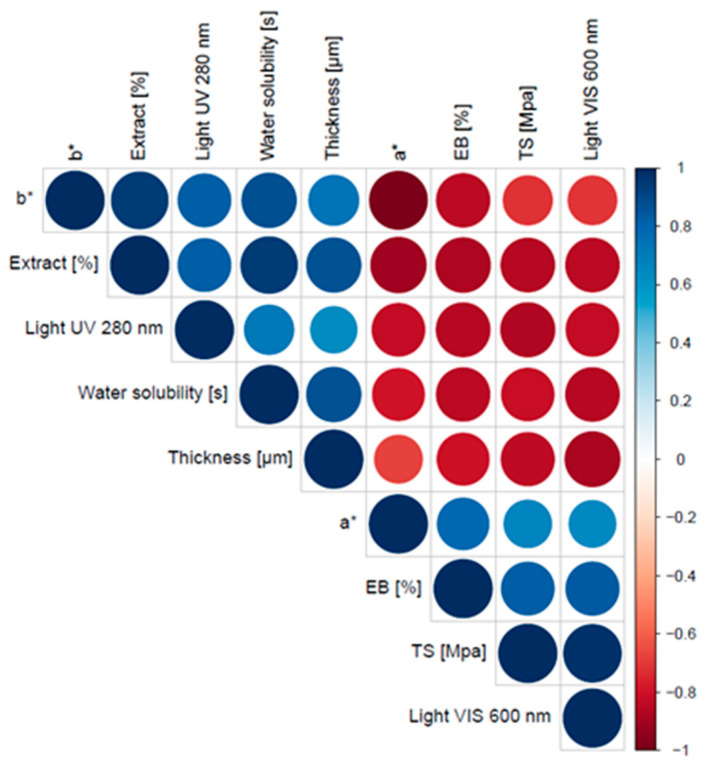
Spearman rank correlation plot based on films’ physical and mechanical profiling. a* and b* are parameters of color (Section 2.4.2.).

In terms of the correlation of the samples, significant positive correlations were observed between the content of propolis extract in the film and its yellow shade, time of water solubility, UV-barrier properties and film thickness (Figure 4). Conversely, there were negative correlations between the propolis extract content in the film and the red tint, mechanical properties and the light transparency of the film. The results suggest that the content of propolis extract in the film had a great influence on its optical and mechanical properties.

### 3.5. Antifungal Activity of Films with Propolis Extract

Table 3 shows DZGI of test fungi by pullulan films containing propolis extracts. The control pullulan film had no antifungal properties (not shown in Table 3). Referring to the classification of the susceptibility of strains to antimicrobial films by Djenane et al. [50], yeasts were less sensitive than molds to films with propolis extracts. Only *R. mucilaginosa* was sensitive to all three EP1 films and DZGI values were between 9.6 and 10.1 mm. In turn, the *C. krusei* and *C. albicans* strains were sensitive to E3/10, and *C. albicans* additionally to E1/10, with DZGI of 9.4 mm, 9.1 mm and 10.2 mm, respectively. The *S. cerevisiae* strain was only sensitive to the EP2/10 film. DZGI of the remaining test yeasts ranged from 6.2 to 8.8 mm. Different sensitivity of the test molds to pullulan films containing propolis extracts was found. DZGI ranged from 6.1 to 16.5 mm and was statistically significantly larger (*p* < 0.05) than DZGI of yeast growth inhibition. Most of the test molds were susceptible to all films, with a few exceptions (DZGI > 9.0 mm, Table 3).

**Figure 5:**
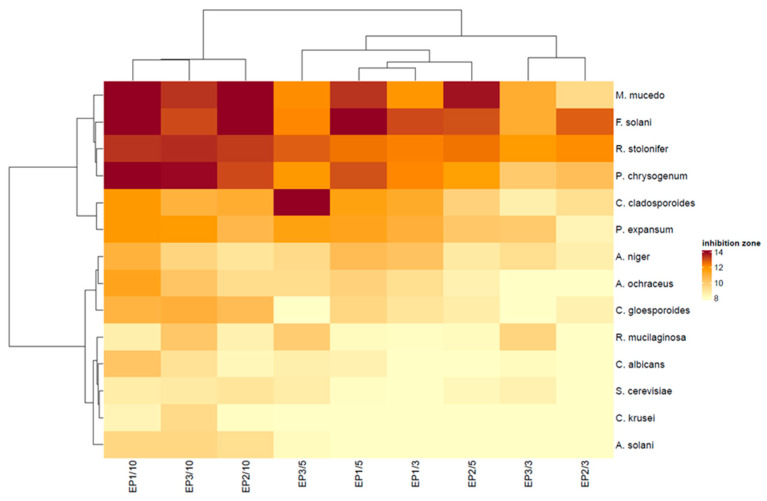
Heat map of antifungal activity of pullulan films with propolis extract (light yellow to dark red corresponding to a progressive increase in the size of the zones of inhibition of growth).

**Figure 6:**
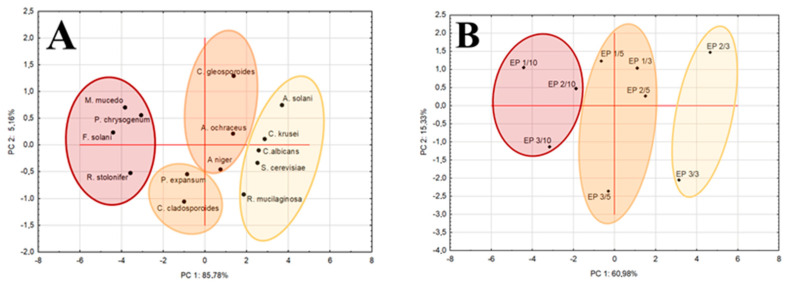
PCA map (**A**,**B**) from PLS-DA analysis based on the diameters of the zones of growth inhibition of the test strains and type of pullulan films with propolis extract.

Analysis of the heat map (Figure 5) and PCA map (Figure 6A) further highlighted differences in detail. The analysis identified four main groups of fungi depending on the size of the zones of growth inhibition. In the group of strains insensitive or poorly sensitive to pullulan films with propolis extract were all test yeast strains and one mold strain (*S. cerevisiae*, *C. krusei*, *C albicans, R. mucilaginosa* and *A. solani*). The moderately sensitive strains include *A. niger, P. expansum* and *C. cladosporioides* as well as *C. gloeosporioides* and *A. ochraceus*, and the most sensitive mold strains are *M. mucedo, P. chrysogenum, F. solani* and *R. stolonifer*.

In our previous study [30], the antifungal activity of propolis extracts from Bochnia (E1), Siedlce (E2) and Ełk (E3) was determined using the microdilution method. The minimum inhibitory concentrations (MIC) of E1 ranged from 2 to 16 mg/mL, E2 from 2 to 8 mg/mL, and E3 from 2 to 32 mg/mL, depending on the strain. These results demonstrate the antifungal potential of propolis extracts. Polish propolis comes mainly from the leaf buds of the black poplar (*Populus nigra*). The regions of Bochnia, Siedlce and Ełk differ slightly in terms of climate and vegetation used by bees. Ełk County is located above the northern limit of black poplar occurrence [70] and white poplar [71]. The lack of these tree species in the Ełk County area may be the reason for a lower content of bioactive components in its chemical composition compared to propolis obtained from the more southerly Bochnia County and Siedlce County regions with a greater frequency of black poplar and white poplar in forests.

Principal component analysis (PCA, Figure 6B) was used to classify the antifungal activity of pullulan films with propolis extract with high accuracy. According to the results of the analysis, three main groups of films were distinguished. Films containing 10% propolis extract, regardless of its origin, were found to have the strongest antifungal activity (E1/10, E2/10 and E3/10). The second group included pullulan films containing 5% propolis extract and a film containing 3% E1 (E1/5, E2/5 and E3/5 and E1/3). Pullulan films with the lowest propolis extract content (EP2/3 and EP3/3) had the weakest antifungal activity. In our previous research, we found that EP1 was richer in flavonoids and the total content of polyphenols than EP2 and EP3 (Section 2.1). EP1 extract also contained more caffeic acid, dimethyl caffeic acid, chrysin, pinocembrin and galangin than EP2 and EP3 [30]. This rich content of bioactive ingredients in EP1 may explain the stronger action of EP1 films against all test fungi, especially EP1/10 (heat map, Figure 5). The mechanism of action of the pullulan film with propolis extract is due to the exertion of an inhibitory and/or fungicidal effect as a result of direct contact of film surface with cells and fungal spores, which results in inhibition of their growth around bioactive films. The components of the propolis extract trapped in the matrix of the pullulan film are gradually released into the environment. Propolis extract destroys the cytoplasmic membrane of fungi, which induces their death [72,73]. Correa et al. [74] suggest that antifungal activity of propolis extracts is probably due to the ability of polyphenols to form complexes with soluble proteins present in fungal cell walls by disrupting chitin synthesis causing loss of integrity. Stahli et al. [75] highlight that the loss of cell wall integrity due to the action of propolis extract reduces the metabolic activity of fungi. On the other hand, it should be noted that the content of propolis extract in the pullulan film had a decisive influence on the antifungal activity of the films, and not the differences in the content of bioactive ingredients in the propolis extract. At the same time, it is related to the physical properties of the films, i.e., film thickness and structure, which influence the optical and mechanical properties.

## 4. Conclusions

Pullulan films with propolis extract and glycerol were successfully prepared using the casting method. The inclusion of propolis extract into pullulan film increased the film thickness and the intensity of the yellow color and extended the dissolution time of the film. Films with propolis extract showed antifungal activity, and films with 10% propolis extract exhibited the strongest inhibitory effects on the growth of fungi (regardless of the origin of propolis). In general, pullulan films with propolis extract can be considered as natural active packaging to protect food against fungi. In the future, we plan to investigate the effectiveness of the pullulan film with propolis extract in food packaging.

## Figures and Tables

**Table 1:** Color parameters (L*, a*, b*) and total color difference (∆E) of pullulan film with propolis extract.

Film Sample	L*	a*	b*	ΔE
Control ^1^	90.98 ± 0.41^d^	−0.47 ± 0.06 ^a^	0.87 ± 0.04 ^a^	1.28 ± 0.39 ^a^
E1/3	90.52 ± 0.30 ^cd^	−0.94 ± 0.05 ^bc^	3.17 ± 0.41 ^bc^	3.11 ± 0.45 ^bc^
E1/5	89.45 ± 0.55 ^ab^	−1.52 ± 0.11 ^d^	5.72 ± 0.60 ^d^	8.12 ± 1.39 ^e^
E1/10	89.67 ± 0.32 ^ab^	−2.05 ± 0.28 ^e^	8.75 ± 1.09 ^e^	9.16 ± 0.46 ^e^
E2/3	90.95 ± 0.25 ^d^	−0.79 ± 0.04 ^b^	2.45 ± 0.16 ^b^	2.26 ± 0.24 ^ab^
E2/5	90.54 ± 0.26 ^cd^	−1.08 ± 0.06 ^c^	3.65 ± 0.26 ^c^	3.53 ± 0.25 ^c^
E2/10	89.32 ± 0.30 ^a^	−1.73 ± 0.24 ^de^	7.78 ± 0.76 ^e^	7.84 ± 0.85 ^e^
E3/3	91.01 ± 0.15 ^d^	−0.82 ± 0.04 ^b^	2.41 ± 0.19 ^b^	2.20 ± 0.22 ^ab^
E3/5	90.46 ± 0.29 ^cd^	−0.93 ± 0.17 ^bc^	3.86 ± 0.74 ^c^	3.73 ± 0.77 ^c^
E3/10	90.04 ± 0.42 ^bc^	−1.45 ± 0.15 ^d^	6.43 ± 0.87 ^d^	6.29 ± 0.95 ^d^

^1^ Control—pullulan film without propolis extract, E1—pullulan films with propolis extract from Bochnia County, E2—pullulan films with propolis extract from Siedlce County, E3—pullulan films with propolis extract from Ełk County. The superscripts different letters in a column indicate significant differences (*p* < 0.05).

**Table 2:** Thickness, moisture content and mechanical parameters of films with propolis extract.

Film Sample	Thickness	Moisture Content	Mechanical Parameters	
	(µm)	(%)	TS (MPa)	EB (%)
Control ^1^	86.0 ± 8.5 ^a^	10.60 ± 0.37 ^a^	24.62 ± 2.12 ^f^	21.00± 0.92 ^c^
E1/3	173.7 ± 4.6 ^bcde^	9.98 ± 0.38 ^a^	14.42 ± 1.99 ^cde^	15.92 ± 1.51 ^b^
E1/5	183.3 ± 25.4 ^bcde^	10.68 ± 0.46 ^a^	13.13 ± 2.44 ^bcd^	12.00 ± 2.78 ^ab^
E1/10	248.8 ± 19.8 ^e^	9.63 ± 0.58 ^a^	8.52 ± 1.36 ^ab^	12.15 ±1.45 ^ab^
E2/3	115.1 ± 23.9 ^abc^	10.62 ± 0.32 ^a^	19.4 ± 1.94 ^e^	14.56 ± 1.15 ^ab^
E2/5	134.7 ± 12.8 ^abc^	9.44 ± 0.12 ^a^	15.46 ± 1.60 ^cde^	14.86 ± 1.10 ^ab^
E2/10	214.08 ± 11.9 ^de^	9.86 ± 0.07 ^a^	12.98 ± 0.61 ^bcd^	11.57 ± 1.08 ^a^
E3/3	102.0 ± 18.2 ^ab^	10.60 ± 0.33 ^a^	16.81 ± 1.71 ^de^	16.80 ± 3.24 ^bc^
E3/5	158.0 ± 14.7 ^abcd^	9.55 ± 0.37 ^a^	10.91± 1.54 ^abc^	15.57 ± 2.53 ^ab^
E3/10	189.3 ± 16.0 ^cde^	9.91 ± 0.54 ^a^	7.66 ± 1.54 ^a^	10.72 ± 1.62 ^a^

^1^ Control—pullulan film without propolis extract, E1—pullulan films with propolis extract from Bochnia County, E2—pullulan films with propolis extract from Siedlce County, E3—pullulan films with propolis extract from Ełk County. The different superscript letters in a column indicate significant differences (*p* < 0.05).

**Table 3:** Diameters of zones of growth inhibition (DZGI) of fungal strains by pullulan films containing propolis extract.

	EP1/3	EP1/5	EP1/10	EP2/3	EP2/5	EP2/10	EP3/3	EP3/5	EP3/10
	(mm ± SD)								
Yeast									
*R. mucilaginosa*	8.1 ± 0.6 ^bcAB^	8.2 ± 0.4 ^bcB^	8.6 ± 0.5 ^aB^	7.3 ± 0.8 ^cA^	8.2 ± 0.5 ^bcB^	8.5 ± 0.6 ^aB^	9.6 ± 0.7 ^efC^	9.9 ± 0.9 ^dC^	10.1 ± 0.8 ^C^
*C. albicans*	7.8 ± 0.6 ^abBC^	8.5 ± 0.7 ^bcCDE^	10.2 ± 0.7 ^bF^	6.2 ± 0.5 ^aA^	7.6 ± 0.7 ^abB^	8.3 ± 0.7 ^aBCDE^	8.2 ± 0.5 ^bcBCD^	8.6 ± 0.5 ^bcDE^	9.1 ± 0.3 ^abE^
*C. krusei*	7.7 ± 0.6 ^abA^	7.9 ± 0.7 ^bAB^	8.4 ± 0.7 ^aB^	7.6 ± 0.5 ^abA^	7.9 ± 0.7 ^bcAB^	8.1 ± 0.7 ^aB^	7.6 ± 0.4 ^abcA^	7.6 ± 0.5 ^abA^	9.4 ± 1.0 ^abC^
*S. cerevisiae*	7.4 ± 0.6 ^abAB^	8.1 ± 0.6 ^bBC^	8.7 ± 0.7 ^aC^	7.2 ± 0.6 ^bcA^	8.3 ± 0.5 ^bcC^	9.0 ± 0.5 ^aC^	8.5 ± 0.7 ^cdC^	8.7 ± 0.7 ^bcC^	8.8 ± 0.5 ^aC^
Mold									
*C. gloeosporioides*	9.0 ± 0.5 ^cBC^	9.5 ± 0.4 ^cdC^	10.8 ± 0.6 ^bcD^	8.5 ± 0.9 ^deB^	8.7 ± 0.5 ^cBC^	10.5 ± 1.1 ^bcD^	6.7 ± 0.2 ^abA^	7.6 ± 0.6 ^aA^	11.0 ± 0.7 ^deD^
*A. solani*	6.1 ± 0.1 ^aA^	6.3 ± 0.1 ^aA^	9.5 ± 0.1 ^abC^	6.1 ± 0.0 ^abcA^	6.4 ± 0.1 ^aA^	9.2 ± 0.1 ^abcBC^	6.2 ± 0.1 ^aA^	8.2 ± 0.8 ^abcB^	9.5 ± 0.6 ^abcC^
*F. solani*	13.1 ± 1.0 ^gB^	14.7 ± 1.0 ^hC^	15.5 ± 1.2 ^eC^	12.8 ± 0.7 ^gB^	13.0 ± 0.6 ^ghB^	14.7 ± 1.0 ^eC^	11.1 ± 0.6 ^gA^	12.2 ± 1.2 ^fgB^	13.1 ± 1.2 ^fgB^
*R. stolonifer*	12.3 ± 1.1 ^fgABC^	12.5 ± 0.5 ^fgABC^	13.4 ± 0.9 ^dBC^	12.1 ± 1.2 ^gBC^	12.5 ± 0.7 ^fgABC^	13.3 ± 1.2 ^dBC^	11.7 ± 0.8 ^gA^	12.8 ± 0.5 ^gABC^	13.5 ± 1.4 ^gC^
*C. cladosporioides*	11.2 ± 0.7 ^defDE^	11.5 ± 0.4 ^efE^	11.8 ± 0.3 ^bcdE^	9.2 ± 0.5 ^deAB^	9.7 ± 0.7 ^deBC^	11.1 ± 1.0 ^cDE^	8.6 ± 0.8^c dA^	10.4 ± 0.3 ^deCD^	10.9 ± 0.6 ^cdeDE^
*A. niger*	10.3 ± 1.1 ^dCD^	10.5 ± 0.8 ^deD^	10.9 ± 0.6 ^bcD^	8.6 ± 0.3 ^deA^	8.8 ± 0.4 ^cdAB^	9.0 ± 0.4 ^aAB^	9.2 ± 0.5 ^deAB^	9.4 ± 0.5 ^bcAB^	9.6 ± 0.3 ^abcdBC^
*A. ochraceus*	9.2 ± 0.6 ^cC^	9.7 ± 0.7 ^dCD^	11.4 ± 1.1 ^bcE^	7.0 ± 0.5 ^abcA^	8.5 ± 0.3 ^bcBC^	9.3 ± 0.6 ^abBCD^	8.0 ± 0.8 ^bcB^	9.3 ± 0.9 ^bcCD^	10.2 ± 1.1 ^bcdeD^
*M. mucedo*	11.9 ± 1.1 ^efBC^	13.4 ± 1.4 ^gC^	15.3 ± 1.6 ^eDE^	9.4 ± 0.9 ^eA^	13.8 ± 1.4 ^hCD^	16.5 ± 1.5 ^fE^	11.1 ± 0.9 ^gB^	12.1 ± 1.2 ^fgBC^	13.4 ± 1.3 ^gC^
*P. expansum*	11.0 ± 0.8 ^deCD^	11.4 ± 0.5 ^efCDE^	11.8 ± 0.5 ^cE^	8.4 ± 0.8 ^dA^	10.1 ± 0.5 ^eB^	10.7 ± 0.4 ^bcBC^	10.0 ± 0.6 ^fB^	11.5 ± 0.6 ^efCDE^	11.7 ± 0.2 ^efDE^
*P. chrysogenum*	12.2 ± 1.1 ^efgCD^	13.0 ± 1.1 ^gCDE^	15.3 ± 1.2 ^eF^	10.4 ± 0.9 ^fAB^	11.6 ± 0.9 ^fBC^	13.1 ± 1.4 ^dDE^	10.0 ± 1.0 ^efA^	11.8 ± 1.0 ^fgC^	13.9 ± 1.0 ^gE^

Control—pullulan film without propolis extract, E1—pullulan films with propolis extract from Bochnia County, E2—pullulan films with propolis extract from Siedlce County, E3—pullulan films with propolis extract from Ełk County. Data are means ± standard deviation (SD); *n* = 3. Means values in the same column with different uppercase letters and in the same row with lowercase letters are significantly different (*p* < 0.05).

## Data Availability

Data available from the corresponding author.
